# Within the Hospitals

**Published:** 1896-04-11

**Authors:** 


### The Institutional Workshop.

### WITHIN THE HOSPITALS.

By Our Special Commissioner.

### DERBYSHIRE ROYAL INFIRMARY.

We made a very careful inspection of these new buildings, as we were anxious to see how they answered when in full work. Plans and a full description of the infirmary will be found in The Hospital for July 21st, 1894, Vol. XVI., page 343. It will be remembered that a novel feature was introduced by the architects, who, following the foreign plan, have laid down the floors in terrazzo or some similar material. It was thought at the time that these composite floors would prove very cold for the patients and the staff. We had an opportunity of gathering the opinion and experience of the medical officers, the matron, the nurses, and the patients, who all state that during the excessive cold of the winter 1894-1895, there was no perceptible difference between the terrazzo floor and the ordinary teak or oak. So far as comfort is concerned, all seem to prefer composite to wood floors. For some reason which we were not able to explain the appearance of the floors left something to be desired, as they were dull and spotty in places, a defect which seemed likely to increase with wear. It is worthy of remark that the material used in the kitchens does not present this defect, but whether this is due to the method of cleansing adopted we were unable to satisfy ourselves. The Secretary explains that the kitchen floors are properly finished while the floors in the wards are not. The contractors have informed the Building Committee that they wish to refinish the ward floors, and this will be done shortly. It is maintained that the present state of the ward floors is due to defective work only. We shall watch the effect of the refinishing with interest, and report it in due course. In any case, the point is an important one, because unless the appearance of the terrazzo floors in the wards at Derby can be materially improved, and some steps taken to prevent them wearing spotty and losing their bright appearance, the experiment must be regarded as a failure. After all, appearance is a material factor in promoting the efficiency of every part of a modern hospital. If, therefore, the terrazzo floor proves in practice to present after slight or continuous wear a faded or shabby appearance it will be deservedly discredited, and we are not likely to see it prevail as a floor substance for ward purposes in British hospitals.

#### The Kitchens and Adjacent Offices.

There can be no question that anyone who wishes to see a model plan for kitchen, larders, and offices should visit the Royal Infirmary, Derby. The whole of these offices are maintained in a high state of efficiency which reflects the greatest credit on the matron and her staff. We never remember to have seen anything better kept, and the whole of the fittings and arrangements are excellent. A practical point of importance is noticeable in the cook's larder and meat store. Although the ceilings, as well as the bottom of the glazed tiles in the lavatories, are rounded off, in the case of the cook's larder and meat store the top glazed bricks are not rounded off, and in consequence the staff cannot clean them thoroughly without damaging the ceiling. At the time of our visit we noticed a black rim on the ceiling and upper tiles, which the Secretary states is not now observable, although they have not been whitened since. We noticed further that the kitchen was very hot, due possibly to the fact that the flues of the gas roaster were not large enough to carry off all the fumes, and the heat from which consequently escaped through the wire gratings which are inserted just below the flues at the top of the roasters and which open into the kitchen itself. Experiments were being made with a view to obviate this overheating in the kitchens, which we are informed have proved entirely successful.

#### Ventilation.

Another practical point is the absence of some cross ventilation in the board-room, and, as a consequence, this room is, we were informed, anything but pleasant during the Christmas festivities, or whenever it contains a large number of people. Self-acting ventilators are also wanting in the linen-rooms and stores. The more we inspect hospitals, the more we are impressed with the multiplicity of detail which it is essential an architect should understand and grasp before he can be said to have mastered the intricacies of hospital construction. It seemed at the first blush that the one feature which would sure to be found excellent in a modern hospital would be its ventilation; but our experience is that the adequate ventilation of every part of a new hospital is very often not provided for.

#### Heating by Steam.

It may be remembered that this hospital is heated throughout by steam, and a reference to our former description will show that much ingenuity was manifested in adopting the system, so as to make it efficient and successful. Despite the great care exercised, we noticed that the arrangements were not entirely satisfactory. A great deal of dirt finds its way through the ventilators. A further point which illustrates the prejudice existing against artificial ventilation in this country is that we found an impression to prevail throughout the hospital that the cold air ventilators were to be kept shut during the summer because no air ought to be admitted through them unless it passed over heated pipes.

It is some months since we made our inspection, and we understand that steps have been taken to remedy most of the matters here referred to. It is well to mention them, however, because they deal with practical points, and so are full of instruction for all who take an interest in modern hospital buildings.

#### The New Nurses' Home.

This home is well conceived, and the details are, on the whole, good. It is a great pity, however, that instead of following the Edinburgh plan and decorating the nurses' rooms on some scheme of colour which provided that every three rooms should be different from all the others, the committee have apparently bought a large amount of blue paper which has been hung in all the rooms on one floor at least, and so tends to produce a monotonous effect which is neither attractive nor satisfactory. The distance of the home from the hospital proper is attended by some disadvantage and risk to the nurses. A covered way for the greater part of the distance has now been provided, which will obviate much of the risk, but the nurses have still to traverse "a comparatively short asphalte path in the open" on their way to and from the nurses' home. A covered way was objected to by the architects on the ground that there is no sort of difficulty in insisting on the nurses being properly clothed and shod during inclement weather. In such a case there would be no more risk to the nurses than is incurred by the visiting staff who live away from the hospital. We are inclined to think, however, that there is this great difference between the visiting staff and the nursing staff—that the latter may often have to pass from their home to the hospital during the hours they are on duty, and that it would be most inconvenient were it necessary for nurses so circumstanced to have to change their shoes and to put on outdoor dress whenever they had to go to the nurses' home. Of course, if a proper robing-room could be provided in the hospital, and arrangements made which would obviate all necessity for any nurse to visit the nurses' home during the hours on duty, the necessity for a covered way would be minimised, if it was not entirely removed.

### The Officers' Quarters.

There is one feature about the Derbyshire Royal Infirmary which must attract and fix the attention of the visitor. The accommodation provided for the resident staff of all grades is excellent. The rooms of the matron, the resident medical and other officers are just what they should be. They exhibit a due sense of the importance of providing rooms of ample proportion, and so arranged as to contribute to the health and comfort of those who have to occupy them. The provision of separate dining halls for various classes of officers, each of which is provided with suitable fittings according to the different grades, is a commendable feature, and reflects credit not only upon the authorities, but upon the architects, to whose foresight these improvements are no doubt largely due. We wish we could bestow the same praise upon the arrangements for residents at other hospitals. Unfortunately the architects, as a rule, very often fail to provide adequately for the comfort of the resident staff. Two striking examples of this are the residents' rooms at St. Thomas's Hospital, London, and the sleeping accommodation for the servants at the Royal Infirmary, Liverpool. The last is about the gravest fault we have seen in a modern hospital plan. We said at the time the new buildings were opened, and we again repeat, that the architect of the Liverpool Royal Infirmary (Mr. Waterhouse, R.A.), who is really responsible for this grave defect, ought, as a matter of professional reputation, to remedy the mischief by providing adequate quarters for the servants, at his own cost.

### DERBYSHIRE HOSPITAL FOR SICK CHILDREN.

In response to a special invitation, we recently paid a second visit to this institution. On entering, it presents a spacious area and a smart appearance, as the whole of the buildings have been redecorated recently. There can be no doubt, however, that the plan is an extravagant one for so small an institution, entailing considerable loss of room and an unnecessary annual expenditure for maintenance. At the time of our visit a children's demonstration by the Sunday schools, churches, and chapels of Derby was in progress. The children paraded the streets with bands of music, and left large quantities of flowers at the Children's Hospital. All the children in the hospital who were able to get up were placed outside in the verandah, and each procession formed up opposite to it, whilst the school children sang hymns for the amusement of the sick little ones. There can be no doubt that these demonstrations do a great deal of good by inducing the young to take an interest in the Children's Hospital, which must re-act upon the parents, and so lead to subscriptions which it would probably be impossible to obtain otherwise. The garden and grounds are well kept, and afford ample space for the sick inmates in fine weather. They are a distinct feature of the institution, and are altogether admirable. We again noticed that the lavatories and offices of this hospital are not in good order or well kept, the bath-rooms and w.c.'s leaving much to desire. This is the more remarkable seeing that the whole institution has recently been redecorated, and the lack of smartness in these places shows a want of oversight in the management which we greatly regret. The honorary lady superintendent, Miss Cupis, who courteously showed us over the institution, has been in charge of it since it was opened, some sixteen years ago. Miss Cupis is genuinely interested in the institution, and assured us that the children have everything they can require or desire, although the cost per bed is so small as to have again called for comment in "Burdett's Hospital and Charities Annual."

